# Mammography in Breast Disease Screening and Diagnosis

**DOI:** 10.3390/jpm13020228

**Published:** 2023-01-27

**Authors:** Daniele Ugo Tari, Fabio Pinto

**Affiliations:** 1 Department of Diagnostic Senology, District 12, Palazzo della Salute, Caserta LHA, 81100 Caserta, Italy; 2 Department of Radiology, “A. Guerriero” Hospital, Caserta LHA, 81025 Marcianise, Italy

Female breasts can suffer from a wide spectrum of pathologies, ranging from inflammatory diseases to benign and malignant tumors. In particular, breast cancer (BC) is the most common cancer in women of all ages, with more than 2 million diagnoses being given every year and a high economic and psychological impact on both the healthcare system and the population [1,2]. Furthermore, women have to face numerous risk factors, such as age and familiarity, nutrition, endogenous and/or exogenous hormonal stimulation, as well as breast density, which represents an independent risk factor to the development of BC [3]. To reduce the risk and harm of these conditions, primary prevention is fundamental, and the importance of early diagnosis has been widely demonstrated [4,5].

Digital mammography (DM) represents the gold standard for breast cancer screening in women aged 40 and over (especially 50–69 years old), with different guidelines proposed around the world [6]. Nevertheless, although screening is considered the main instrument to achieve early diagnosis, the effectiveness of its current modalities is highly debated due to the presence of false-positive and false-negative cases, interval cancers, and overdiagnosis, which are associated with discomfort and/or potential psychological harm to the patients [7].

Digital Breast Tomosynthesis (DBT) is emerging as the standard of care for breast imaging based on improvements in both screening and diagnostic imaging outcomes [8]. The additional information obtained from tomosynthesis acquisition decreases the confounding effect of overlapping tissue, allowing for improved lesion detection, characterization, and localization. Actually, several prospective studies support the use of DBT as an adjunct or alternative to standard imaging techniques due to the increase in the detection rate up from 0.5 to 2.7 per 1000 screened women [9]. At the same time, there is still no statistically significant and clinically relevant reduction in the interval cancer rate, so more studies are needed to consolidate the wide use of DBT in a population-based screening program [10].

Contrast-enhanced spectral mammography (CESM) is the most recent imaging tool. CESM uses iodinated contrast to reveal areas of increased blood supply within the breast. In particular, after intravenous administration of contrast, low-energy and high-energy images are retrieved in one acquisition using a dual-energy technique, and a recombined image is constructed that enables the visualization of the area of contrast uptake. Consequently, CESM could be considered as an alternative to magnetic resonance imaging (MRI) that is easier to perform in daily clinical practice due to its costs and better compliance from patients. Its main indication would be the assessment of screening recalls, but the correlation with DM is mandatory if used as a single-assessment tool [11]. Further studies, more evidence-based interpretation guidelines for benign–malignant criteria, and AI-based CAD technology might increase the usage of CESM as an important primary imaging tool in the assessment for screening recalls [12].

DM and DBT could be used as guidance for vacuum-assisted breast biopsy (VABB). In particular, studies have shown improved targeting and sampling of non-calcified lesions (asymmetries, masses, and architectural distortion) with DBT-guided biopsy in comparison with DM-guided stereotactic biopsy [13]. Consequently, the use of a DBT for this type of interventional technique can potentially reduce patient discomfort and radiation exposure without affecting clinical outcomes. Additionally, in their meta-analysis, Cullinane et al. [14] suggested that it is reasonable to perform VABB as definitive treatment for certain B3 lesions (specifically LN, FEA, radial scar, and papillary lesions), while surgical excision should continue as the mainstay of treatment for ADH.

All these evaluations about the use of DM and its advancements strengthens the concept that breast imaging for screening and diagnosis is a challenging area that requires deep training. Personalized screening and multimodality approaches are the solution to guarantee to the patients a complete and early diagnosis, especially in these difficult times where the pandemic has created long-term and permanent challenges that will change the practice of medicine in the future [15,16]. The redistribution of clinical activities and the fear of contracting the infection for both patients and healthcare workers (HCWs) have had and will continue to have significant socio-economic and psychological impacts, especially on breast cancer screening, where time is fundamental and the impact on the entire community is relevant. Consequently, the needs of modifying the standard diagnostic–therapeutic care pathways (DTCP), in accordance with the increased use of telemedicine, will raise new medico-legal implications for both patients and the health system.

Trying to deepen and clarify all these aspects is mandatory to face all these new challenges with commitment and dedication.
